# CXCL16/CXCR6 Axis in Adipocytes Differentiated from Human Adipose Derived Mesenchymal Stem Cells Regulates Macrophage Polarization

**DOI:** 10.3390/cells10123410

**Published:** 2021-12-03

**Authors:** Seung-Cheol Lee, Yoo-Jung Lee, Inho Choi, Min Kim, Jung-Suk Sung

**Affiliations:** 1 Department of Life Science, Dongguk University-Seoul, Goyang 10326, Korea; hen3420@dongguk.edu (S.-C.L.); wjdyd85@naver.com (Y.-J.L.); pipikimmin@dongguk.edu (M.K.); 2 Department of Pharmaceutical Engineering, Hoseo University, Asan 31499, Korea; inhochoi@hoseo.edu

**Keywords:** hADMSCs, adipocytes, cell–cell interaction, chemokines, CXCL16/CXCR6 axis, macrophage polarization

## Abstract

Adipocytes interact with adipose tissue macrophages (ATMs) that exist as a form of M2 macrophage in healthy adipose tissue and are polarized into M1 macrophages upon cellular stress. ATMs regulate adipose tissue inflammation by secreting cytokines, adipokines, and chemokines. CXC-motif receptor 6 (CXCR6) is the chemokine receptor and interactions with its specific ligand CXC-motif chemokine ligand 16 (CXCL16) modulate the migratory capacities of human adipose-derived mesenchymal stem cells (hADMSCs). CXCR6 is highly expressed on differentiated adipocytes that are non-migratory cells. To evaluate the underlying mechanisms of CXCR6 in adipocytes, THP-1 human monocytes that can be polarized into M1 or M2 macrophages were co-cultured with adipocytes. As results, expression levels of the M1 polarization-inducing factor were decreased, while those of the M2 polarization-inducing factor were significantly increased in differentiated adipocytes in a co-cultured environment with additional CXCL16 treatment. After CXCL16 treatment, the anti-inflammatory factors, including p38 MAPK ad ERK1/2, were upregulated, while the pro-inflammatory pathway mediated by Akt and NF-κB was downregulated in adipocytes in a co-cultured environment. These results revealed that the CXCL16/CXCR6 axis in adipocytes regulates M1 or M2 polarization and displays an immunosuppressive effect by modulating pro-inflammatory or anti-inflammatory pathways. Our results may provide an insight into a potential target as a regulator of the immune response via the CXCL16/CXCR6 axis in adipocytes.

## 1. Introduction

Adipose tissue, which is generally recognized as a complex metabolic organ, produces various factors which have important endocrine functions, including cytokines, chemokines, and adipokines, such as leptin and adiponectin [1,2]. Of these factors, circulating inflammatory chemokines are strongly associated with obesity, which is a state of chronic low-grade systemic inflammation, and the development of allergic or immune diseases as a result of their interaction with macrophages [3,4]. In particular, adipose tissue macrophages (ATMs) play a crucial role in crosstalk with adipocytes by modulating their localization and inflammatory features [5,6,7]. ATMs can be classified into M1 or M2 macrophages, which are formed via the influence of the various innate and adaptive immune cells involved in the regulation of adipose tissue inflammation under different adipocyte conditions [6,8,9,10]. Classically activated M1 macrophages are stimulated by Th1 cells, CD8 cells, and mast cells secreting interferon-δ (IFN-δ), tumor necrosis factor-α (TNF-α), interleukin-6 (IL-6), and C–C motif chemokine ligand 2 (CCL2). They contribute to the inflammatory response of neighboring cells, particularly in adipocytes displaying a crown-like dead structure [11,12]. In contrast, alternatively activated M2 macrophages are distributed in healthy adipose tissue via the expression of anti-inflammatory factors, such as fibronectin, cluster of differentiation 206 (CD 206), C–C motif chemokine ligand 22 (CCL22), and interleukin-10 (IL-10). They are induced by Th2 cytokines, which are involved in the repair or remodeling of adipose tissue [13,14,15]. Research into M1/M2 paradigm has provided a useful framework with which to alter adipocyte function as an inflammatory effector because macrophages are considered to be key regulatory cells in the control of adipose tissue inflammation via the secretion of cytokines and chemokines [16,17,18].

Chemokine receptors are G protein-coupled receptors (GPCR) classified into four classes and named according to the ligand they interact with [19]. The regulation of chemokine receptors and their downstream signaling pathways are considered promising molecular targets since they modulate various biological functions, such as cell migration, cancer metastasis, and angiogenesis [20]. Among various chemokine receptors, CXC-motif receptor 6 (CXCR6) is known to induce the recruitment of T cells that participate in chronic inflammation, the invasion of glial precursor cells, and in the migration of mesenchymal stem cells via the interaction of its specific ligand CXC-motif chemokine ligand 16 (CXCL16) [21,22,23].

Human mesenchymal stem cells, first discovered in non-hematopoietic stem cell populations, are multipotent stem cells that differentiate into osteoblasts, chondrocytes, neuronal cells, and adipocytes [24]. They migrate into injured tissue in response to signals sent in certain in physiological circumstances and differentiate into local components of injured tissue, secreting various types of growth factors and cytokines [25,26]. It has widely been known that the homing and migration of mesenchymal stem cells are mediated by various cytokines, growth factors, and chemokines upon interaction with their receptors, including CXCR6 [22,27]. While the functional role of the CXCL16/CXCR6 axis on the migratory capacity is intensively studied in mesenchymal stem cells that tend to migrate into certain tissues as part of cellular responses, the function of CXCR6 on differentiated adipocytes that tend to remain in their original location has yet to be elucidated [28].

Our previous report showed that CXCR6 is highly expressed in adipocytes differentiated from human adipose derived mesenchymal stem cells (hADMSCs) [29]. In this study, we further evaluated and clarified the role of CXCR6 in adipocytes interacting with macrophages and its underlying molecular mechanisms.

Here, differentiated adipocytes expressing CXCR6 were co-cultured with THP-1 cells, which were polarized into M1 or M2 macrophages, with or without additional CXCL16 treatment, which is the specific ligand of CXCR6. It is important to determine whether adipocytes interact with macrophages by expressing pro-inflammatory or anti-inflammatory factors on the molecular level in response to additional CXCL16 treatment, since focusing on the crosstalk between the chemokines and the macrophages in adipose tissue can aid in the understanding of the mechanisms associated with the modulatory effects of adipose tissue inflammation [30,31,32,33]. 

## 2. Materials and Methods

### 2.1. Cell Cultures and the Adipogenic Differentiation of hADMSCs

The human mesenchymal cell line, hADMSCs, were purchased from CEFO (Seoul, Korea) and cultured in hADMSC growth medium (CEFO, Seoul, Korea) until cells were 80% confluent. To induce adipogenic differentiation, hADMSCs of biological passage number 4 were used. Cells were detached using Accutase (Innovative Cell Technologies, San Diego, CA, USA) and then seeded into appropriate cell culture dishes at the density of 6000 cells/cm^2^ and cultured until they become 80% confluent. Then, differentiation into adipocytes was induced by culturing the cells for 18 days in high-glucose Dulbecco’s Modified Eagle Medium (DMEM) (Gibco, Amarillo, TX, USA) containing 1% penicillin streptomycin (Gibco, Amarillo, TX, USA), 10% fetal bovine serum (Alphabioregen, Boston, MA, USA), 1 μM dexamethasone (Sigma-Aldrich Chemical, St. Louis, MO, USA), 100 μM indomethacin (Sigma-Aldrich Chemical, St. Louis, MO, USA), 10 μg/mL insulin (Welgene, Gyeongsan, Korea), and 500 μM 3-isobutyl-1-methylxanthine (Sigma-Aldrich Chemical, St. Louis, MO, USA). The differentiation medium was replaced every 3 days. Differentiated adipocytes were validated by the relative expression of mature adipocyte markers and the stained cellular lipid droplets in differentiated adipocytes were compared with hADMSCs (Figure S1).

### 2.2. RNA Extraction and Real-Time PCR

RNA was isolated using Trizol (Invitrogen, Waltham, MA, USA), following the manufacturer’s protocol. cDNA was synthesized from 0.8 µg of total RNA using Reverse Transcription Master Premix (ELPIS, Daejeon, Korea) according to the manufacturer’s instructions. To amplify the cDNA, 40 cycles of PCR were performed using SYBR Green PCR Master Mix (KAPA Biosystem, Wilmington, MA, USA), according to the manufacturer’s protocol. The expression levels of each target gene were normalized to GAPDH as an internal control. The specific oligonucleotide primers used in the study are listed in Table 1.

### 2.3. Western Blot

The differentiated adipocytes were collected using an RIPA buffer (Biosolution, Seoul, Korea) containing a protease or phosphatase inhibitor (Sigma-Aldrich Chemical, St. Louis, MO, USA) and then centrifuged at 16,200 rpm for 30 min. Cell extracts were quantified using a BCA protein assay kit (Pierce Biotechnology, Waltham, MA, USA), according to the manufacturer’s instructions. Approximately 30–40 µg of protein was separated using 10% sodium dodecyl sulfate polyacrylamide gel electrophoresis (SDS-PAGE) and transferred to polyvinlylidene difluoride membranes (GE Healthcare, Chicago, IL, USA) for western blot analysis. At first, the transferred membrane was blocked with 1X Tris-buffered saline with Tween 20 (Sigma-Aldrich Chemical, St. Louis, MO, USA) containing 5% skimmed milk (BD Biosciences, Franklin Lakes, NJ, USA) for 1 h and then incubated with the primary antibodies p-Akt, t-Akt, p-p38, t-p38, p-JNK, t-JNK, and t-ERK1/2 (Cell Signaling Technology, Danvers, MA, USA), and p-NF-κB, t-NF-κB, and p-ERK1/2 (Santa Cruz Biotechnology, Santa Cruz, CA, USA) at appropriate dilutions in 1X TBST containing 1% skimmed milk overnight at 4 °C. The membrane was washed three times with 1X TBST for 10 min and incubated with secondary anti-rabbit (Cell Signaling Technology, Danvers, MA, USA) and anti-mouse (Santa Cruz Biotechnology, Santa Cruz, CA, USA) antibodies in 1× TBST containing 1% skimmed milk for 45 min. The membrane was also washed three times for 15 min and the images were visualized using an enhanced chemiluminescence detection reagent (GE Healthcare, Chicago, IL, USA) and a Chemi-Doc (Bio-Rad Laboratories, Hercules, CA, USA) imaging device.

### 2.4. Enzyme-Linked Immunosorbent Assay (ELISA)

Secretion levels of soluble TNF-α and IL-10 in co-cultured THP-1 cells and differentiated adipocytes with CXCL16 treatment were determined using human TNF-α and an IL-10 ELISA kit (BioLegend, San Diego, CA, USA), according to the manufacturer’s instructions.

### 2.5. THP-1 Cell Differentiation

THP-1 human monocytes were purchased from ATCC (Manassas, VA, USA) and cultured in RPMI1640 (Welgene, Gyeongsan, Korea) containing 10% FBS, 2 mM L-glutamine, and 1% penicillin streptomycin. THP-1 cells were differentiated into M0 macrophages using phorbol 12-myristate 13-acetate (Sigma-Aldrich Chemical, St. Louis, MO, USA) for 24 h and then polarized to M1 or M2 macrophages using 20 ng/mL of LPS (Sigma-Aldrich Chemical, St. Louis, MO, USA) or 20 ng/mL of IL-4 and IL-13 (PeproTech, Rocky Hill, NJ, USA), respectively, for 48 h.

### 2.6. Polarization of THP-1 Cells Co-Cultured with Differentiated Adipocytes

THP-1 human monocytes were seeded in a 0.4 μm transwell insert (Corning Inc, Corning, NY, USA) at a density of 3 × 10^5^ cells/well with PMA and then added to a 6-well plate with differentiated adipocytes with or without CXCL16 treatment for 24 h. M0 macrophages co-cultured with adipocytes were polarized into M1 or M2 macrophages with LPS or IL-4 and IL-13 treatment, respectively, w/wo CXCL16 treatment for 48 h. The M0, M1, and M2 macrophages were extracted to evaluate the levels of gene expression exhibited by M1 or M2 polarization markers with additional CXCL16 treatment.

### 2.7. Oil Red O Staining of Differentiated Adipocytes

Cells were washed twice with PBS and fixed with 10% formalin (Sigma-Aldrich Chemical, St. Louis, MO, USA) for 10 min on ice. After fixation, cells were washed with PBS and 60% isopropanol (Sigma-Aldrich Chemical, St. Louis, MO, USA) and air dried. Cells were stained with Oil Red O (Sigma-Aldrich Chemical, St. Louis, MO, USA) at room temperature for 30 min and were washed twice with distilled water. Stained lipid droplets were imaged using microscopy (Nikon, Tokyo, Japan). To quantify the relative lipid contents in cells, 100% isopropanol was used to dissolve the dye and the absorbance was measured at 492 nm with a microplate reader (Tecan, Männedorf, Switzerland).

### 2.8. Statistical Analysis

The statistical analysis was performed using GraphPad Prism 5.0 (GraphPad Software Inc., San Diego, CA, USA). The results were expressed as the mean ± standard error of at least three independent experiments. Multiple comparisons were conducted using a one-way ANOVA with the Bonferroni method. Comparisons between two samples were made using by *t*-tests.

## 3. Results

### 3.1. Validation of THP-1 Cell Polarization into M1 or M2 Macrophages

Under different conditions, ATMs can polarize into M1 or M2 macrophages. In lean adipose tissue, M2 ATMs promote adipocyte survival leading to adipose tissue hyperplasia and the regulation of insulin sensitivity by interacting with adipocytes to maintain energy homeostasis. In contrast, M1 ATMs, which are induced by oxidative stress, trigger an inflammatory response in neighboring cells in obese adipose tissue [8,9,13]. 

**Figure 1:**
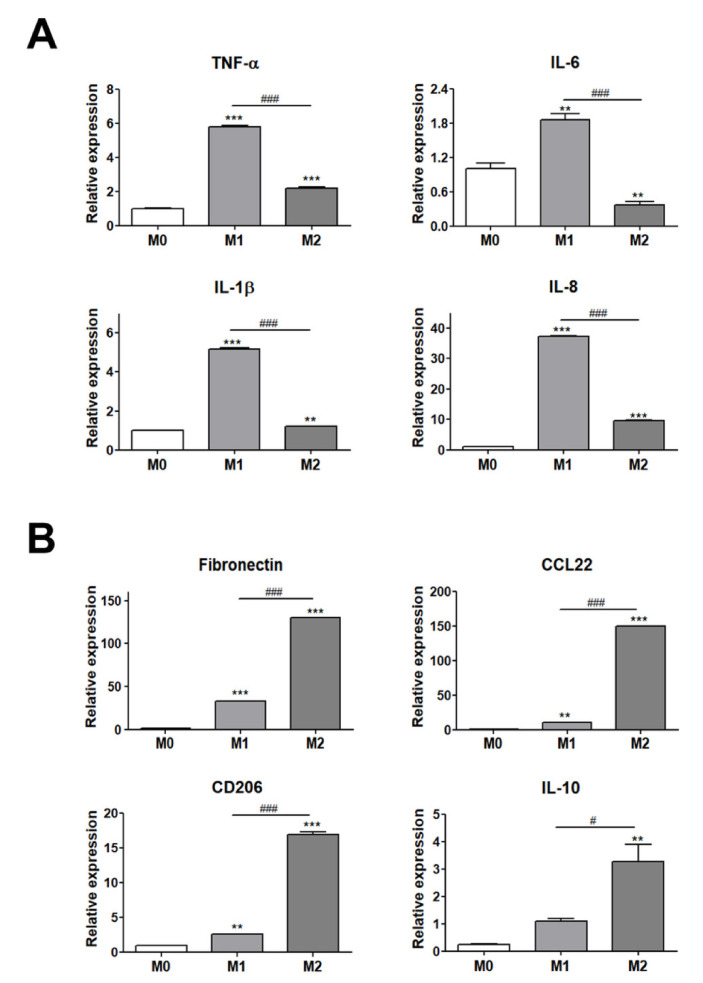
Validation of THP-1 cell differentiation to M1 or M2 macrophages. (**A**) Gene expression levels of M1 polarization markers (TNF-α, IL-6, IL-1β, and IL-8) and (**B**) M2 polarization markers (fibronectin, CD206, IL-10, and CCL22). ** *p* < 0.01 and *** *p* < 0.001 indicate statistically significant differences compared with the M0 group. *n* = 4 trials per samples and control. ^#^ *p* < 0.05 and ^###^ *p* < 0.001 indicate statistically significant differences between M1 and M2 macrophages.

To evaluate the interaction between adipocytes and ATMs, we used THP-1 human monocytes that express specific markers in a similar way to freshly isolated cells and that can polarize into M1 or M2 macrophages by LPS and IL-4 and IL-13 treatment, respectively [34,35]. The polarization of THP-1 cells into M1 or M2 macrophages was validated by assessing the gene expression of M1 and M2 polarization-specific markers using real-time RT-PCR (Figure 1). Human monocyte THP-1 cells were differentiated into M0 macrophages with PMA treatment for 24 h and these were then polarized to M1 or M2 macrophages with a treatment of 20 ng/mL LPS or 20 ng/mL IL-4 and IL-13, respectively, for 48 h. The levels of gene expression for the M1 and M2 macrophage markers were evaluated for the validation of THP-1 cell polarization into M1 or M2 macrophages. The expression levels of M1 macrophage markers (TNF-α, IL-1β, IL-6, and IL-8) were significantly higher in M1 macrophages with LPS treatment as compared with M2 macrophages (Figure 1A). In addition, the levels of gene expression for the M2 macrophage markers (fibronectin, CCL22, CD206, and IL-10) were significantly upregulated in M2 macrophages with IL-4 and IL-13 treatment as compared with M1 macrophages (Figure 1B). Based on these results, THP-1 differentiation into M0, M1, and M2 macrophages was validated for further experiments.

### 3.2. Expression Levels of M1 and M2 Polarization Markers in THP-1 Cells Co-Cultured with Differentiated Adipocytes upon Additional CXCL16 Treatment

To examine whether adipocytes interact with THP-1 cells and affect M1 or M2 polarization by CXCL16 treatment, THP-1 cells were co-cultured with adipocytes differentiated from hADMSCs and then differentiated into M0, M1, and M2 macrophages. 

**Figure 2:**
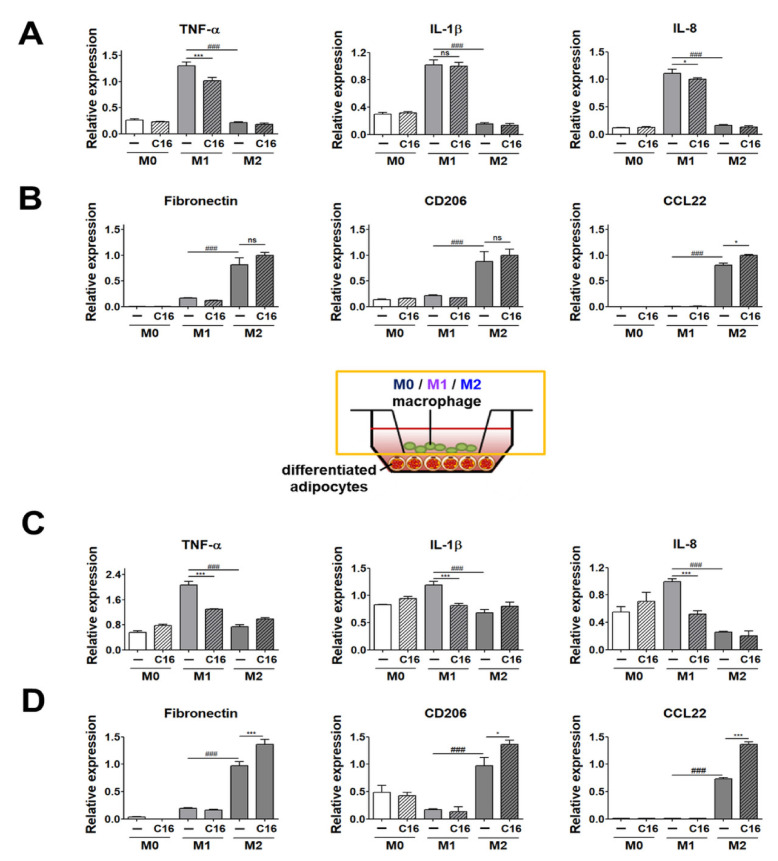
Expression levels of M1 and M2 polarization markers in THP-1 cells co-cultured with differentiated adipocytes upon additional CXCL16 treatment. (**A**) Gene expression levels of M1 polarization markers (TNF-α, IL-1β, and IL-8) and (**B**) M2 polarization markers (fibronectin, CD206, and CCL22) in THP-1 cells upon CXCL16 treatment. *n* = 3 trials per samples and control. (**C**) Gene expression levels of M1 polarization markers (TNF-α, IL-1β, and IL-8) and (**D**) M2 polarization markers (fibronectin, CD206, and CCL22) in THP-1 cells co-cultured with differentiated adipocytes upon additional CXCL16 treatment. *n* = 5 trials per samples and control. * *p* < 0.05 and *** *p* < 0.001 compared with macrophages without CXCL16 treatment. ^###^ *p* < 0.001 indicate statistically significant differences between M1 and M2 macrophages. Not significant, ns.

We first measured the expression levels of M1 and M2 polarization markers in M0, M1, and M2 macrophages upon CXCL16 treatment. As shown in Figure 2A, the expression levels of M1 polarization markers (TNF-α, IL-8) were slightly decreased. Notably, the expression levels of M2 polarization markers (CCL22) were slightly increased in M2 macrophages from THP-1 cells with additional CXCL16 treatment (Figure 2B). The expression levels of fibronectin and CD206 were also slightly increased, but this was not statistically significant (Figure 2B). These results indicate that additional CXCL16 treatment on THP-1 cells attenuates polarization into M1 macrophages and enhances polarization into M2 macrophages on its own. 

We then examined the expression levels of M1 and M2 polarization markers in THP-1 cells co-cultured with differentiated adipocytes with additional CXCL16 treatment. The expression levels of the M1 macrophages markers (TNF-α, IL-1β, and IL-8) were significantly decreased with additional CXCL16 treatment (Figure 2C). In contrast, the expression levels of the M2 macrophage markers (fibronectin, CD206, and CCL22) were significantly increased with additional CXCL16 treatment (Figure 2D).

### 3.3. Expression Levels of M1 and M2 Polarization-Inducing Factors on Differentiated Adipocytes upon Additional CXCL16 Treatment

The gene expression levels of M1-inducing factors (CCL2, IL-6, and TNF-α) and M2-inducing factors (M-CSF, IL-10, and IL-13) were evaluated in differentiated adipocytes co-cultured with THP-1 cells to examine whether the expression of M1- and M2-inducing factors in co-cultured adipocytes affected M1 or M2 polarization upon additional CXCL16 treatment. 

**Figure 3:**
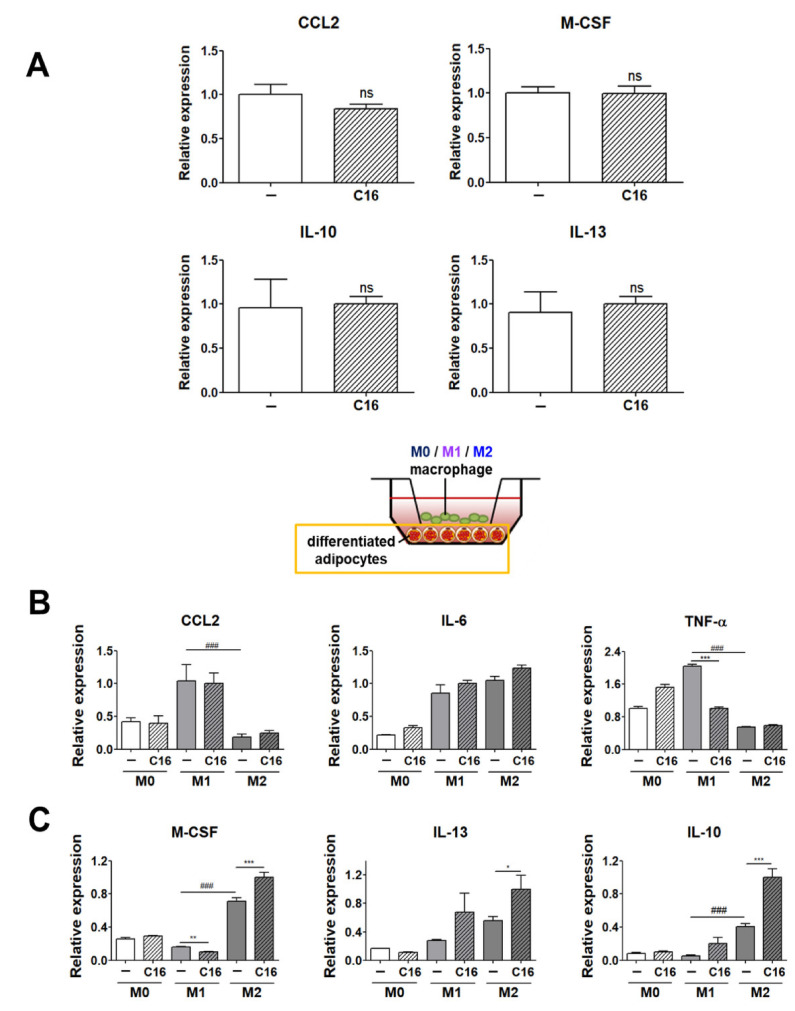
Expression levels of M1 and M2 polarization-inducing factors on differentiated adipocytes upon additional CXCL16 treatment. (**A**) Gene expression levels of M1 polarization-inducing factors (CCL2) and M2 polarization-inducing factors (M-CSF, IL-10, and IL-13) in adipocytes upon CXCL16 treatment. *n* = 3 trials per samples and control. (**B**) Gene expression levels of M1 polarization-inducing factors (CCL2, IL-6, and TNF-α) and (**C**) M2 polarization-inducing factors (M-CSF, IL-10, and IL-13) in differentiated adipocytes co-cultured with THP-1 cells upon additional CXCL16 treatment. *n* = 5 trials per samples and control. * *p* < 0.05, ** *p* < 0.01 and *** *p* < 0.001 compared with adipocytes without CXCL16 treatment. ^###^ *p* < 0.001 indicate statistically significant differences between adipocytes co-cultured with M1 or M2 macrophages. Not significant, ns.

We analyzed the levels of M1 and M2 polarization-inducing factors in differentiated adipocytes in the environment without THP-1 cells. As shown in Figure 3A, there was no significant difference in the expression levels (CCL2, M-CSF, IL-10, and IL-13) of M1 or M2 polarization-inducing factors in differentiated adipocytes in the absence of THP-1 cells upon CXCL16 treatment. This indicates that CXCL16 treatment of adipocytes cultured without macrophages does not display a potential to affect the M1 or M2 polarization of THP-1 cells. 

However, interestingly, the expression levels of M1 or M2 polarization-inducing factors in differentiated adipocytes were highly regulated in the co-culture environment with THP-1 cells that had been polarized into M1 or M2 macrophages (Figure 3B,C). The expression levels of M1 polarization-inducing factors (IL-6 and TNF-α) were significantly decreased (Figure 3B), while those of M2 polarization-inducing factors (M-CSF, IL-10, and IL-13) in differentiated adipocytes significantly increased with additional CXCL16 treatment (Figure 3C). 

### 3.4. The Secretion Levels of TNF-α and IL-10 in Cell Culture Media of THP-1 Cells and THP-1 Cells Co-Cultured with Differentiated Adipocytes

**Figure 4:**
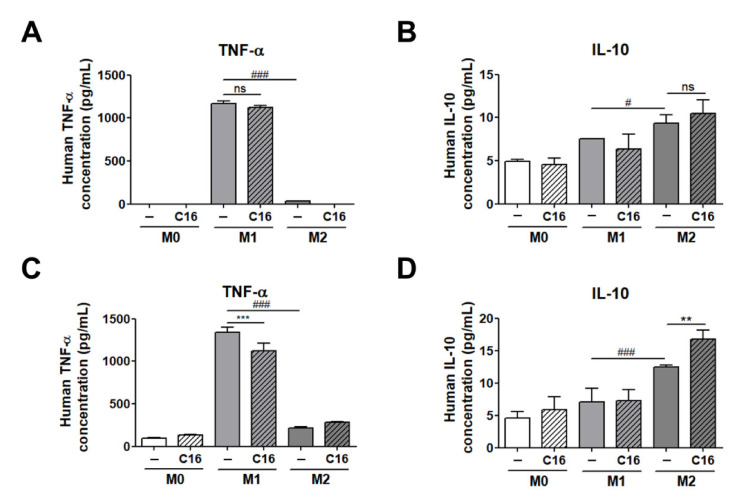
The secretion levels of TNF-α and IL-10 in cell culture media of THP-1 cells and THP-1 cells co-cultured with differentiated adipocytes. (**A**) Secretion levels of TNF-α and (**B**) IL-10 in a cell culture medium in THP-1 cells and the secretion levels of (**C**) TNF-α and (**D**) IL-10 in a cell culture medium of co-cultured THP-1 cells upon additional CXCL16 treatment. *n* = 5 trials per samples and control. ** *p* < 0.01 and *** *p* < 0.001 compared with samples without CXCL16 treatment. ^#^ *p* < 0.05 and ^###^ *p* < 0.001 indicate statistically significant differences between M1 and M2 macrophages. Not significant, ns.

We examined the secretion levels of the M1 and M2 polarization-inducing factors (TNF-α, IL-10), both of which exhibited significant differences in expression levels in adipocytes co-cultured with THP-1 cells (Figure 4). 

As shown in Figure 4A,B, the secretion level of TNF-α and IL-10 in cell culture media were not differed with additional CXCL16 treatment in M1 and M2 macrophages from THP-1 cells. Notably, the secretion levels of TNF-α and IL-10 in a co-cultured media of differentiated adipocytes and THP-1 cells were significantly decreased and increased, respectively, consistent with the data shown in Figure 3B,C (Figure 4C,D). These results suggest that CXCL16 interacts with CXCR6, which is highly expressed in differentiated adipocytes, and then regulates the polarization of THP-1 cells into M1 or M2 macrophages via the secretion of factors including TNF-α and IL-10.

### 3.5. Inflammatory Signaling Pathways in Differentiated Adipocytes Co-Cultured with THP-1 Cells upon Additional CXCL16 Treatment

**Figure 5:**
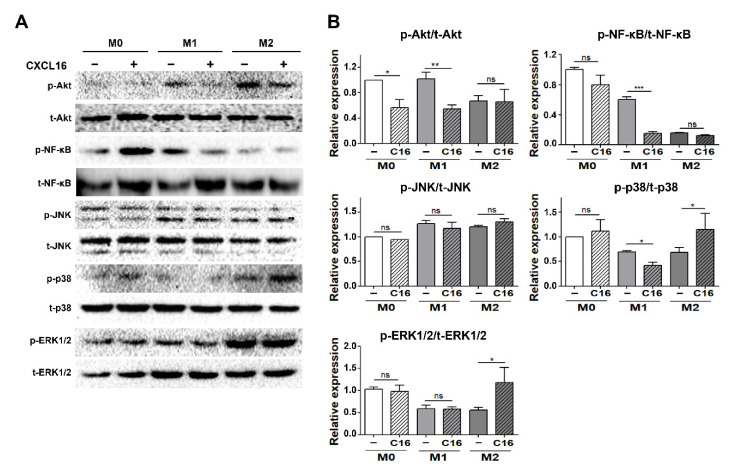
Inflammatory signaling pathways in differentiated adipocytes co-cultured with THP-1 cells upon additional CXCL16 treatment. (**A**) Pro-inflammatory (Akt, NF-κB) and anti-inflammatory pathways (JNK, p38, and ERK1/2) in adipocytes co-cultured with THP-1 cells following additional CXCL16 treatment for 30 min. (**B**) Relative protein intensity of p-Akt/t-Akt, p-NF-κB/t-NF-κB, p-JNK/t-JNK, p-p38/t-p38, and p-ERK1/2/t-ERK1/2 in adipocytes. *n* = 3 trials per samples and control. * *p* < 0.05, ** *p* < 0.01, and *** *p* < 0.001 indicate statistically significant differences. Not significant, ns.

To evaluate which inflammatory signaling pathways are regulated in differentiated adipocytes in a co-culture environment with THP-1 cells, the expression of signaling proteins mediating the mitogen-activated protein kinase (MAPK) and nuclear factor kappa-light-chain-enhancer of activated B cells (NF-κB) signaling pathways were examined (Figure 5). The nuclear factor NF-κB pathway has long been considered a pro-inflammatory signaling pathway and the MAPK pathway serves as an anti-inflammatory signal that suppresses the expression of NF-κB-dependent inflammatory genes for anti-inflammatory purposes [36,37,38].

The activation of the Akt/NF-κB pathway significantly decreased in differentiated adipocytes in the presence of THP-1 cells polarized into M1 macrophages with additional CXCL16 treatment. In contrast, ERK1/2 and p38 MAPK, but not JNK, were activated in differentiated adipocytes in the presence of THP-1 cells polarized into M2 macrophages.

## 4. Discussion

Previously, we reported that CXCR6 was highly expressed on cell surfaces during the differentiation of hADMSCs into adipocytes [29]. Despite the discovery on the regulatory function of CXCR6 expression and the function of the CXCL16/CXCR6 axis in the migratory capacity of hADMSCs, the function of the CXCL16/CXCR6 axis in adipocytes with poor migratory capacities has been insufficiently studied [22,28]. Adipocytes secrete various growth factors, cytokines, and chemokines, which are related to the metabolic syndrome, insulin resistance, obesity-related disease, and immune responses [5,39,40,41]. They can interact with immune cells, especially ATMs that are differentially located, depending on the state of adipose tissue [8,11,12]. Under different conditions, ATMs can polarize into M1 or M2 macrophages. In lean adipose tissue, M2 ATMs promote adipocyte survival leading to adipose tissue hyperplasia and the regulation of insulin sensitivity by interacting with adipocytes to maintain energy homeostasis. In contrast, M1 ATMs, which are induced by oxidative stress, trigger an inflammatory response in neighboring cells in obese adipose tissue [8,9,13]. 

To evaluate the interaction between adipocytes and ATMs, the polarization of THP-1 cells into M1 or M2 macrophages was first validated by assessing the gene expression of M1 and M2 polarization markers (Figure 1). Gene expression levels of M1 polarization markers (IL-1β) and M2 polarization markers (fibronectin and CD206) showed no significant difference in THP-1 cells with additional CXCL16 treatment (Figure 2A,B). Additionally, the gene expression levels of M1 markers (TNF-α and IL-8) and M2 markers (CCL22) were only slightly downregulated or upregulated, respectively, with CXCL16 treatment. Recent research has reported that a specific motif of CXCR6 expressed in THP-1 cells binds with CXCL16 and affects the recruitment or adhesion of leukocytes [42]. This suggests that CXCR6 in THP-1 cells may have the potential to regulate the expression of inflammatory factors through its interaction with CXCL16. Therefore, the slight modulation of M1 and M2 polarization of THP-1 cells following CXCL16 treatment can be interpreted as being due to the CXCL16/CXCR6 axis. 

On the other hand, interestingly, the expression levels of M1 and M2 polarization markers in M1 and M2 macrophages were significantly downregulated or upregulated, respectively, upon CXCL16 treatment when they were co-cultured with differentiated adipocytes (Figure 2C,D). 

These results suggest that adipocytes with CXCL16 treatment in co-cultured environments with THP-1 cells can affect the polarization potential of THP-1 cells into M1 and M2 macrophages.

Gene expression levels of M1-inducing factors (CCL2, IL-6, and TNF-α) and M2-inducing factors (M-CSF, IL-10, and IL-13) in differentiated adipocytes co-cultured with THP-1 cells were measured to examine whether the expression of M1- and M2-inducing factors in co-cultured adipocytes affected M1 or M2 polarization with additional CXCL16 treatment. As shown in Figure 3A, there was no statistically significant difference in the levels of CCL2, M-CSF, IL-10, and IL-13, which are basically expressed in adipocytes in physiological conditions upon CXCL16 treatment. However, interestingly, the expression levels of these factors differed significantly upon additional CXCL16 treatment when differentiated adipocytes were co-cultured with THP-1 cells that are polarized into M1 and M2 macrophages. The M1-inducing factors IL-6 and TNF-α decreased upon CXCL16 treatment and the M2-inducing factors M-CSF, IL-10, and IL-13 significantly increased upon CXCL16 treatment in adipocytes co-cultured with THP-1 cells (Figure 3B,C). This suggests that CXCL16 treatment does not affect the expression of M1 or M2 inducing factors in differentiated adipocytes on its own but attenuates polarization into M1 macrophages and promotes polarization into M2 macrophages when in the co-cultured environment of adipocytes and THP-1 cells. That is, adipocytes at the location available to interact with macrophages are significantly affected by CXCL16, resulting in the modulatory expression of M1 or M2 inducing factors.

Measurement of the secretion levels of TNF-α and IL-10 demonstrated that they were significantly modulated in differentiated adipocytes co-cultured with THP-1 cells by additional CXCL16 treatment. Consistent with the results shown in Figure 3, secretion levels of TNF-α and IL-10 were significantly downregulated and upregulated, respectively, in co-cultured media of differentiated adipocytes and THP-1 cells (Figure 4C,D). These results suggest that CXCL16 interacts with CXCR6, which is highly expressed in differentiated adipocytes, and then regulates the polarization of THP-1 cells into M1 or M2 macrophages. 

The MAPK pathway—p38 and ERK1/2 in particular—serves as an anti-inflammatory signal that suppresses the expression of NF-κB-dependent inflammatory genes and makes them potential targets for anti-inflammatory activity [36,37]. The NF-κB pathway is a pro-inflammatory signaling pathway that involves the expression of pro-inflammatory genes, including cytokines, chemokines, and adhesion molecules [38]. It was found in this study that the anti-inflammatory pathway (p38MAPK, ERK1/2) was significantly activated by CXCL16 treatment in differentiated adipocytes co-cultured with THP-1 cells that had been polarized into M2 macrophages. In contrast, activation of the pro-inflammatory pathway (Akt, NF-κB) decreased with additional CXCL16 treatment when co-cultured with THP-1 cells that were polarized into M1 macrophages (Figure 5A,B). 

M2 macrophages are known to produce higher levels of immunosuppressive cytokines compared to M1 macrophages, and studies showed that mesenchymal stem cells promoting M2 macrophage polarization display immunosuppressive effects [43,44,45,46]. On the basis of our observations, we can conclude that CXCL16 can modulate the characteristics of two cell populations via expression of M1 or M2 inducing factors, simultaneously modulating anti- or pro-inflammatory pathways in adipocytes during the interaction between adipocytes and macrophages. This indicates that CXCL16 acts as an anti-inflammatory chemokine via interaction with CXCR6, attenuating the polarization of THP-1 cells into M1 macrophages and enhancing their polarization into M2 macrophages.

Recent studies have revealed that tumor-associated macrophages (TAMs) have been shown to express an M2-like phenotype promoting tumor progression and the metastasis of cancers by upregulating CXCR6 expression [47,48]. In spite of the evidence in these studies supporting the regulatory function of macrophages on CXCR6 expression in cancer cells, the information on the function and the regulatory mechanism of CXCL16/CXCR6 in adipocytes is lacking. Therefore, further comprehensive analysis of the expression level of CXCR6 on cell surfaces and the regulation of signal transduction mediated by the CXCL16/CXCR6 axis in co-cultured environments possesses great potential. Furthermore, the discovery of the regulatory mechanism underlying the role of CXCR6 expression and the regulation of the CXCL16/CXCR6 axis would provide insights into adipose tissue microenvironments.

In summary, our results clarified the role of CXCR6, which is highly expressed in adipocytes differentiated from hADMSCs. It was found that CXCR6 interacts with CXCL16 and subsequently attenuates or enhances the pro-inflammatory or anti-inflammatory response of macrophages in the presence of adipocytes via the Akt, NF-kB or p38, and ERK1/2 pathways. We first demonstrated that CXCL16 plays a crucial role in regulating M1 or M2 polarization via interaction with neighboring ATMs. This finding promises to shed light on the development of a novel therapeutics that could regulate inflammatory responses via CXCL16/CXCR6 interaction in adipocytes with macrophages.

## 5. Conclusions

In this study, we investigated the role of CXCR6 in differentiated adipocytes in interaction with macrophages after CXCL16 treatment. In a co-cultured environment of THP-1 cells with adipocytes, polarization into M1 and M2 macrophages was attenuated and enhanced, respectively, with additional CXCL16 treatment. The polarization into M1 and M2 macrophages was significantly regulated by the expression of M1- and M2-inducing factors in differentiated adipocytes co-cultured with THP-1 cells with additional CXCL16 treatment, demonstrating that the polarization of THP-1 cells into M1 or M2 macrophages was affected by the interaction between CXCL16 and CXCR6 expressed in differentiated adipocytes. Moreover, the pro-inflammatory and anti-inflammatory pathways were modulated by CXCL16 treatment in adipocytes co-cultured with THP-1 cells. In summary, we have shown that the CXCL16/CXCR6 axis in adipocytes functions as the regulator of the inflammatory response via interactions with neighboring macrophages. These results may contribute to development of a novel stem cell therapy in the search for a potential drug target that can regulate the immune response of macrophages via theCXCL16/CXCR6 axis in adipocytes.

## Figures and Tables

**Table 1:** List of oligonucleotide primers used in the study.

Gene Name		Sequences
*TNF-α*	Forward Reverse	AGAGAAGCCAACTACAGACC CAGTATGTGAGAGGAAGAGAA
*IL-6*	Forward Reverse	CAGAACAGATTTGAGAGTAGTGA CGCAGAATGAGATGAGTTGT
*IL-1β*	Forward Reverse	GGCTTATTACAGTGGCAATG TAGTGGTGGTCGGAGATT
*IL-8*	Forward Reverse	GAAGGAACCATCTCACTGT CCACTCTCAATCACTCTCA
*Fibronectin*	Forward Reverse	TCATCCGTGGTTGTATCA GTGGTCTCAGTAGCATCT
*CCL22*	Forward Reverse	AAGGCAGTTACATATCAATACAG GAGGCAGAGGCTTCAATA
*CD206*	Forward Reverse	CGGAGTAGTCATCATTGTG CGAGTGTTCATTCTGTTCA
*IL-10*	Forward Reverse	AAGCCTTGTCTGAGATGAT CCTTGATGTCTGGGTCTT
*CCL2*	Forward Reverse	CGAGAGGCTGAGACTAAC GAAGGTGGCTGCTATGAG
*M-CSF*	Forward Reverse	GAAGGAGGACCAGCAAGT CAGCAAGACCAGGATGAC
*IL-13*	Forward Reverse	ATCCGATCCTCAATCCTC CTGGTTCTGGGTGATGTT
*FABP4*	Forward Reverse	TCAAGAGCACCATAACCTT TTCCACCACCAGTTTATCA
*Adiponectin*	Forward Reverse	ACCACTATGATGGCTCCACT GGTGAAGAGCATAGCCTTGT
*PPARγ*	Forward Reverse	CGAAGACATTCCATTCACAA CACAGACACGACATTCAAT
*GAPDH*	Forward Reverse	TATGACAACAGCCTCAAGAT GAGTCCTTCCACGATACC

## Data Availability

The data presented in this study are available on request from the corresponding author.

## Supplementary Materials

The following are available online at www.mdpi.com/2073-4409/10/12/3410/s1, Figure S1: Validation of adipogenic differentiation of adipocytes from hADMSCs.
