# Supplementary material for: CXCL16/CXCR6 Axis in Adipocytes Differentiated from Human Adipose Derived Mesenchymal Stem Cells Regulates Macrophage Polarization
Source: Cells. 2021 Dec 3;10(12):3410. doi: 10.3390/cells10123410 (PMC8699853; doi:10.3390/cells10123410)
Supplement: Supplementary file 1 [file cells-10-03410-s001.zip › cells-1468740-supplementary.pdf]

Supplementary figure and figure legend

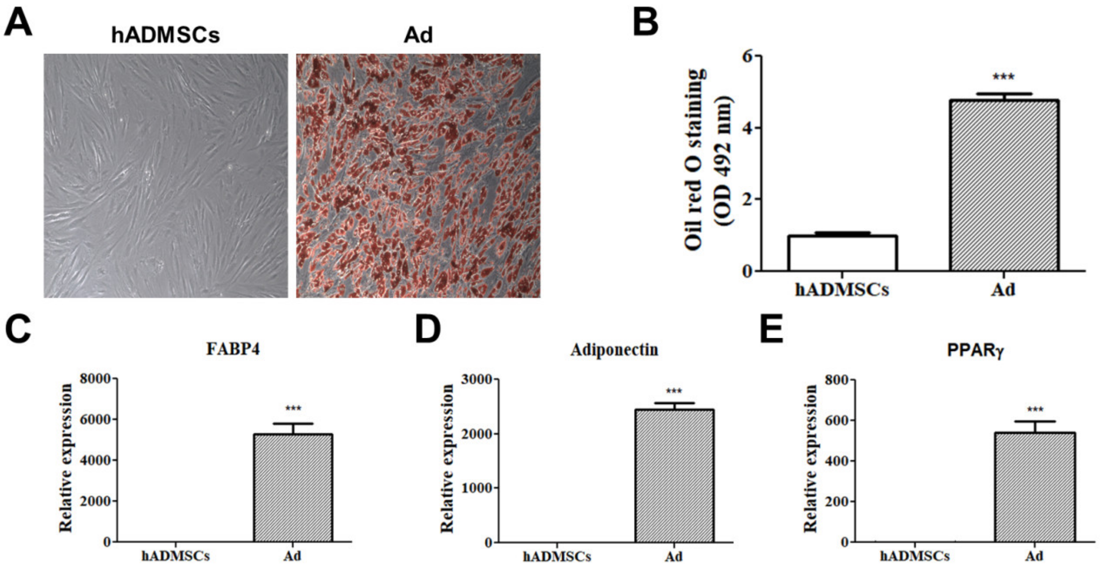

Figure S1. Validation of adipogenic differentiation of adipocytes from hADMSCs. (**A**) ORO-stained lipid droplets in differentiated adipocytes and hADMSCs. (**B**) Relative quantification of lipid droplets in differentiated Adipocytes and hADMSCs. $n = 4$ trial per samples and control. Gene expression levels of adipogenic differentiation markers (**C**) FABP4, (**D**) Adiponectin, and (**E**) PPARγ on hADMSCs and differentiated adipocytes. $n = 3$ trials per samples and control. *** $p < 0.001$ indicates statistically significant compared with the hADMSCs group.
